# Mode splitting in optical microcavities for speckle-free wavelength reconstruction

**DOI:** 10.1038/s41377-025-02073-9

**Published:** 2026-01-01

**Authors:** Ivan Saetchnikov, Elina Tcherniavskaia, Andreas Ostendorf, Anton Saetchnikov

**Affiliations:** 1 Radio Physics Department, Belarusian State University, Minsk, Belarus; 2 Physics Department, Belarusian State University, Minsk, Belarus; 3 Chair of Applied Laser Technologies, Ruhr University Bochum, Bochum, Germany

**Keywords:** Imaging and sensing, Photonic devices, Micro-optics, Optical sensors, Microresonators

## Abstract

Accurate wavelength measurement is critical for spectroscopy, optical communications, semiconductor manufacturing, and quantum research. Emerging reconstructive wavemeters are compact, cost-effective devices that utilize pseudo-random wavelength patterns and computational techniques to provide high-resolution, broadband alternatives to solutions based on frequency beating and interferometry. We propose a novel reconstructive wavemeter that synergizes the advantages of both approaches. Its physical model is based on the integration of thousands of high-quality-factor optical microcavities, which are deformed to stimulate whispering gallery mode splitting. For realizing a wavelength interpreter, we developed a hybrid machine learning approach utilizing boosting methods and variational autoencoders. This enabled the implementation of wavelength interpretation as a rigorous regression task for the first time. The introduced novel concept ensures the uniqueness of the wavelength patterns up to ultra-wide (~100 nm) spectral window while guarantees high (~100 fm) intrinsic sensitivity. The latter allocates the proposed solution right next to the ultimate reconstructive wavemeters based on integrating spheres, but with less calibration efforts, featuring superior miniaturization options and chip-scale integrability.

## Introduction

Precise wavelength measurement, critical for scientific and industrial applications, relies on wavemeters. Widely used in spectroscopy, metrology, and optical calibration^1,2^, wavemeters became essential also for quantum research, where precise wavelength control for quantum information, computing, and sensing^3–5^, is enabled with high-accuracy instruments based on optical heterodyne/beating detection or interferometry^6^. Advances in frequency combs enable optical beating with ~ fm accuracy over tens of nanometers^7^. Electro-optic combs achieve sub-fm resolution but require precise control of pump laser parameters and laser-cavity detuning^8,9^. Interferometry-based wavemeters are constructed on different schemes, with Fizeau leading, thanks to a simple setup, broadband detection, real-time measurement, and sub-10 fm resolution^10^. Whereas optical beating wavemeters require a pump laser, interferometry-based schemes remain bulky, expensive, and alignment-sensitive. This highlights the need for compact, passive solutions combining precision with broadband capability.

Computational methods to reconstruct wavelengths from pseudo-random (speckle) patterns enable high precision in compact devices with enhanced dynamic range^11,12^. Speckle is obtained by directing the light through a disordered medium such as multimode fiber^13–16^, photonic crystal cavity array^17,18^, diffuser/scatterer^19,20^, or integrating sphere^21,22^. The Pearson correlation coefficient effectively assesses speckle-based wavemeters. Among them, the integrating spheres achieve the highest intrinsic sensitivity (sub-100 fm), surpassing multimode fibers by an order of magnitude^23^. A common interpreter linking patterns to wavelengths is the transmission matrix (TM)^14,20,24,25^. The TM approach assumes uncorrelated speckle patterns, which fails at ≲ pm separations, requiring advanced techniques like unsupervised^22,26^ or supervised machine learning (ML)^15,16,19^. Here, wavelength identification has been considered as an ML classification task in narrow windows, reaching resolutions from ~ pm to sub-fm with speckle preprocessing under specific constraints.

Whispering gallery mode (WGM) microresonator is capable of synergizing the advantages of different approaches. This is an interferometer with high quality- (Q-)factors^27,28^ and spectral features defined by the environment and microcavity itself, which enables high-precision sensing^29–31^. Appropriate material and maintenance of well-controlled fabrication conditions turn WGM cavity into a frequency comb source^8,9^. At the same time, inherent discrepancy between microresonators and surface inhomogeneities transform the microscale cavity into a scattering medium with long optical paths (≳100 m), generating unique spectral patterns. Microcavities can produce temporal speckles via laser frequency modulation, enabling ~ fm resolution when combined with ML models incorporating TM-calculated responses^32^. However, the WGM pattern, confined to resonance peaks with spectral gaps and periodicity, limits wavelength determination to a narrow range (~pm). Tracking multiple microcavities extends the range^33–35^, but remains inferior to other physical principles. In weakly deformed spherical microcavities, the circular modes propagating in different equatorial planes lose degeneracy and split with periodicity determined by the cavity eccentricity^36,37^. This is commonly used for the detection of undesirable eccentricity^38,39^. Inspired by the frequency combs, we intentionally induced controllable deformation of optical microspheres to excite/promote mode splitting and to obtain a comb that does not require strict fabrication conditions or excitation lasers.

**Fig. 1:**
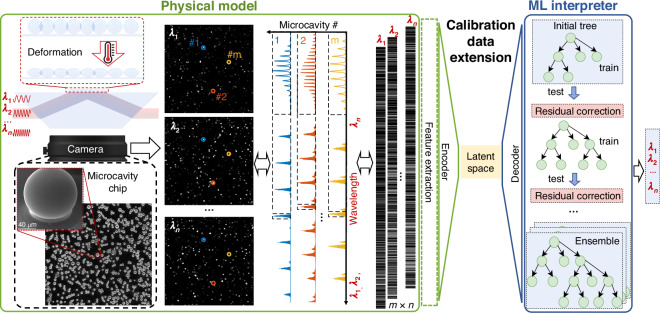
A concept of compact, speckle-free, and broadband reconstructive wavemeter. It combines a novel physical model based on the chip containing thousands of deformed high-Q microresonators with eccentricity mode splitting for the generation of unique wavelength patterns, together with an ML interpreter. The latter includes a unique ensemble of feature extraction, a variational autoencoder, and a regression model based on boosting methods for high high-precision wavemeter in the ultra-wide spectral regions with limited calibration data. The latter consists of repeating broadband WGM spectral responses obtained by tuning a narrow-linewidth laser, which enables operation of the device in passive mode after interpreter training

A novel physical model using a chip with high-Q microresonators with mode splitting realizes the compact, speckle-free, and broadband reconstructive wavemeter with intrinsic sensitivity of ~100 fm. It leverages multiplexed microresonator imaging to generate unique wavelength patterns by simultaneous probing of thousands of optical microresonators, which we previously introduced for sensing^40–44^. The obtained intrinsic sensitivity puts this model alongside the most accurate reconstructive wavemeters based on integrating spheres, but with improved integrability. The unique combination of the microresonator chip featuring eccentricity mode splitting as the physical model, together with a novel ML interpreter that includes feature extraction, variational autoencoders (VAE), and the regression model based on Light Gradient-Boosting Machine (LGBM), paves the way for a high-precision wavemeter in the ultra-wide spectral regions up to ~100 nm (Fig. 1).

## Results

### Eccentricity stimulation in microspheres

**Fig. 2:**
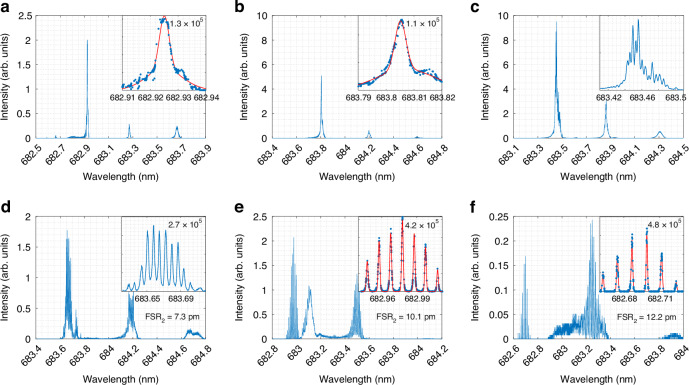
Stimulation of the microsphere eccentricity. Eccentricity mode splitting for representative PMMA microresonator in the initial state (0 °C) (**a**) and heated at 80 °C (**b**), 120 °C (**c**), 140 °C (**d**), 160 °C (**e**), and 180 °C (**f**). Blue lines and dots represent experimental results, red lines - fitting of the resonances with Voigt profile. Insets focus on the most prominent mode

First, we studied mode splitting and its properties using the multiplexed microresonator chips with glass microspheres (Section S1). Despite manufacturer specifying up to 15% non-spherical microresonators, we observed spectrally-resolved mode splitting in no more than 1% of glass microresonators. A similar ratio was found for PMMA microspheres and can be explained by the overlapping of the split modes and resultant resonance broadening (Fig. S1 in Supplementary Information). Unlike the glass with its superior mechanical, thermal and chemical stability, PMMA can be manipulated, e.g., by heating above glass transition temperature (≈100 °C). Thanks to the permanent bonding of the microresonators with the chip substrate and the necessity for subtle deformations only, eccentricity is obtained by placing the PMMA chips on the hot plate without applying external force. The direction of the cavity deformation is perpendicular to the chip and the obtained eccentricity level can be controlled via processing time and temperature. We found that for temperatures below 80 °C the WGM alterations remain negligible. At 220 °C the microresonators deform significantly so that high radiation losses prevent resonance observation, and at 250 °C they melted completely. Effect of the processing time for temperature range [80:180] °C was found negligible and thus processing for 10 min was used further. Comparison of the WGM properties before (0 °C) and after thermal treatment with varying temperature from 80 to 180 °C is shown in Fig. 2.

Processing the chip at 80 °C only broadens the WGM and increases its intensity (Fig. 2b), while at 120 °C side peaks appear but remain poorly resolved, and the resonance intensity peaks are approximately five times the original signal (Fig. 2c). These results indicate that thermal processing also affects the WGM coupling efficiency. This is caused by the thermal response of the low refractive index layer that changes the distance between the microresonators and the coupling unit and thus the coupling strength. The latter being initially optimized for the chip as a whole (see Multiplexed microresonator chip) either improves or degrades depending on the individual microcavity. For the demonstrated cavity the resonance intensity increases 5 times when processing at 120 °C which indicates approaching the critical coupling conditions. At 140 °C, the split modes become more distinct with a pitch of 7.3 pm (≈0.5% eccentricity), but still overlap via side tails, while the mode peak intensities return to the original level and the Q-factor doubles (Fig. 2d). Abrupt resonance intensity drop between spectra shown in Fig. 2c and d is caused by the mode degeneracy violation. Splitted modes became resolved without overlaps with *FSR*2 = 10.1 pm (≈0.7% eccentricity) and Q-factor rises further up to 4.2 × 10^5^ via processing at 160 °C (Fig. 2e). Enhancement of the Q-factor compared to the original state (Fig. 2a) is related to initial small non-sphericity causing overlap of split modes. Further deformations widen the spectral pitch up to 12.2 pm and the mode prominence reduces already by an order of magnitude, indicating vanishing of the resonance signal (Fig. 2f). It vanishes completely for all microcavities with additional processing. High-order modes also exhibit thermal changes, though these can deviate from the dominant mode’s behavior. This results in a mix of spectrally resolved and broadened mode-splitting combs within FSR. As results, the temperature range of 140–160 °C allows to expand the portion of the microresonators with spectrally resolved mode splitting from ≈ 1% up to ≈ 45%.

### Performance assessment

**Fig. 3:**
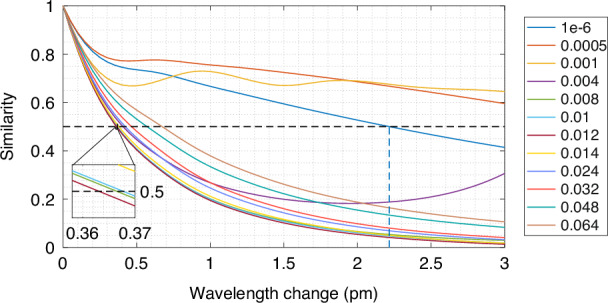
Benchmarking of the wavemeter model. Similarity as a function of the wavelength change for the multiplexed microresonators chip with 200 cavities and different splitting levels (*e* ∈ [1 × 10^−6^, 6.4 × 10^−2^])

We used intrinsic sensitivity to benchmark our model among the other wavemeter designs^23^. Figure 3 representes similarity (Pearson correlation coefficient) of the simulated patterns (details in Section S2) over the wavelength change for different eccentricity values (*e*).

Each curve is calculated as the median value over different wavelength positions, averaged over all repetitions. For a system with 200 non-deformed microresonators, the intrinsic sensitivity is 2.2 pm (half-width at half-maximum,^17^) and compares to multimode fiber-based speckle wavemeters. Slight deformations (*e* = 0.0005) lower sensitivity and at *e* = 0.001 the correlation curve exhibits oscillations due to closely spaced peaks. Further deformations first improve and then degrade the sensitivity, with optimal performance at *e* = 0.012 (≈360 fm). It approaches the accuracy of the integrating spheres, the most precise physical principle for speckle wavemeters currently available. The regularity of splitting peaks can lead to multiple crossings of the 0.5 similarity level when observing a broader wavelength range. This is evident for *e* = 0.004, where correlation begins to rise after a ≈ 2 pm spectral shift. Remarkably, the curve for *e* = 1 × 10^−6^ does not reflect the perfect similarity expected for a limited amount of microresonators. Consequently, the probability of exceeding the 0.5 similarity level decreases with the number of microresonators that we analyze further with a supervised regression model for wavelength reconstruction in the full spectral window.

**Fig. 4:**
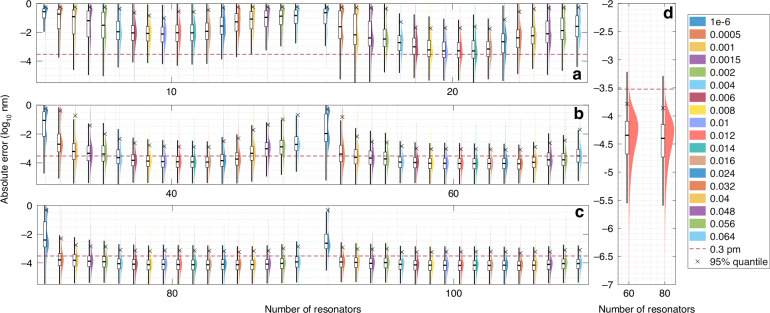
Wavelength prediction error across splitting levels and microresonator numbers. Statistics on the wavelength prediction accuracy in the form of the absolute error value for the simulated data out of 98 measurement points with spectra for different splitting levels (*e* ∈ [1 × 10^−6^, 6.4 × 10^−2^]) and varying number of microresonators in the range of [10:100] that were utilized to build the regression model (**a**–**c**). Red horizontal dashed line indicates the spectral resolution of simulations (0.3 pm). Black crosses show the 95% quantile of the corresponding distributions. **d** The prediction accuracy for the spectral data for *e* = 0.012, which was obtained by accounting for 2352 measurement points

We selected ensemble of decision-trees (LightBoost) as a regressor and for the first time implemented regression model for reconstructive wavemeter (details in Section S3). For performance comparison across eccentricity levels, the same model architecture is used for all simulated patterns assuming that the best accuracy cannot be achieved with the simplest architecture. The dataset without mode splitting (*e* = 1 × 10^−6^) was chosen for optimization, as it is overloaded with spectrally uncertain regions and requires higher model complexity. The optimal values for maximum depth and number of leaves have been determined as 30 and 3584, respectively. The other parameters were set as following: minimum data-in-leaf = 20, learning rate = 0.01, L1 = 1.72 × 10^−8^, L2 = 5.33 × 10^−8^ (see Section S3). We ranged the number of microresonators from 10 to 100, and for each established 30 subsets by randomly selecting from 200 cavities. To maintain reasonable tuning times, we limited the number of repetitions to 98. Performance of the regression model-based wavelength reconstruction for simulated patterns is presented in Fig. 4.

Increasing the number of microresonators improves the reconstruction precision. For patterns without mode splitting (1 × 10^−6^), the absolute error distribution is initially dominated by values ~0.1 nm. As the number of microresonators increases, the distribution evolves to exhibit two maxima (Fig. 4a-c). For 60 microspheres, one maximum appears at ≈0.5 nm and the other at ≈5 pm. Two-maxima distribution is also characteristic of a small number of microresonators with split spectra. Independent on the microresonators’s number the response with eccentricity-induced splitting delivers better prediction accuracy than with sharp peaks. Here, we observed the existence of an optimal splitting level (0.012) that yields the highest accuracy with the fewest microresonators accounted. This agrees perfectly with the correlation model provided above for a limited wavelength range of 3 pm (Fig. 3). Optimal splitting attains peak performance with 60–80 resonators, where the 95% error quantile is 0.6 pm, twice the spectral resolution. Considering the full dataset (2352 repetitions) with *e* = 0.012 for 60–80 microresonators, median prediction accuracy reaches 40 fm, with 95% quantile at 0.1 pm (Fig. 4d). This ensures reliable identification of all wavelengths at the given spectral resolution.

### Experimental validation

Two sets of wavelength patterns were collected from a chip with 5563 microspheres: one from the original chip and one after post-thermal treatment at 160 °C. We limited the prediction range to 1.2 nm and measurement repetitions to 2352 to align with simulation results. To ensure robust statistical analysis, we generated subsets with varying numbers of microspheres (0-100) by randomly selecting from all cavities, with 30 subsets per configuration. Due to inherent assumptions in the simulation model, the optimal LightBoost parameters derived from simulations may not ensure peak performance for the experimental responses. Therefore, the model settings were extra optimized with learning rate = 0.1, the maximum depth of 30, number of leaves = 2560, and regularization parameters L1 and L2 set to 1.14 × 10^−6^ and 2.16 × 10^−8^, respectively (details in Section S3).

**Fig. 5:**
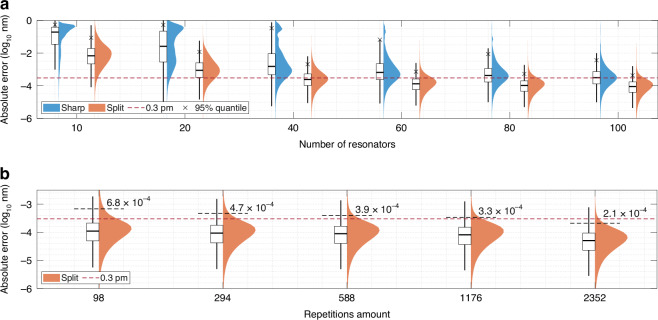
Results on the experimental wavelength prediction accuracy, represented as absolute error values. **a** Errors obtained from 2352 measurement points for the chip with varying number of microresonators in the range of [10:100]: in its original (sharp) and thermally-treated (split) states. **b** Influence of the number of experimental repetitions on the wavelength prediction accuracy within a narrow spectral band for 200 microresonators

The results for experimental wavelength reconstruction agree well with data obtained from simulation (Fig. 5). Split resonances outperform the sharp ones independently on the amount of cavities considered and wavelength detection accuracy improves with resonators’ number (Fig. 5a). When a small number of microcavities with sharp resonances are used, the absolute error distribution exhibits multiple peaks, including one around ~100 pm level. These outliers are observed even for 100 microresonators with sharp peaks. In contrast, the regression model trained on mode-splitting responses exhibits normal distribution in the logarithmic scale, with accuracy improving steadily as the number of microresonators increases. With 40 microresonators exhibiting mode splitting, the median error drops below the wavelength resolution (0.3 pm). For sets with 40 and 60 microresonators, the disparity in prediction accuracy between split and sharp spectra is the most pronounced, reaching two orders of magnitude. With the number of resonators with the split spectra up to 100, the median accuracy reaches the level of 80 fm with 95% quantile at 0.4 pm. This outperforms the wavemeter on sharp spectra by at least one order of magnitude.

Prediction accuracy and reliability in data-driven methods relies on high-quality, consistent, and sufficient training data to enhance reliability, mitigate noise, and effectively capture underlying patterns. Expanding the number of repetitions from 98 to 2352 for the simulated data refines performance by a factor of 6 (Fig. 4). The results for the wavelength prediction accuracy within 1.2 nm spectral region with 200 microresonators and different number of experimental spectra repetitions (from 98 to 2352) available are given in Fig. 5b. When accounting 2352 instead of 98 repetitions, the median error value improves twice, down to 50 fm level, whereas the 95% quantile level reduces from 680 fm down to 210 fm (below the spectral resolution).

### Broadband wavelength prediction

Collecting experimental repetitions for a 1.2 nm range takes hours. Expanding to ~10 nm demands already days or weeks of efforts. Therefore, we propose data augmentation, where experimental repetitions are generated via a variational autoencoder, VAE. This deep learning model effectively allocates complex data to the latent domain, and then reconstructs it in the original domain to reveal key features. Unlike autoencoders, VAEs inject extra stochasticity into the latent description. This enables the generation of new physically plausible samples and makes them effective in data augmentation. It consists of two mirrored deep neural networks (encoder and decoder) to transform input into a 2000-dimensional latent space and then reconstruct the original data. VAE includes five modules, each of convolutional and transposed convolutional layers with LeakyReLU activations (details in Sec. S4). VAE explores the initial distribution, whereas Kullback-Leibler divergence regularizes latent space to a normal distribution, ensuring ultra-precise but realistic reconstruction without merely duplicating originals. To prevent VAE overfitting and capture the full range of factors, the broadband (10.13 nm) dataset was measured 41 times for a chip with 5563 microspheres and a resolution of 0.3 pm (33765 points).

**Fig. 6:**
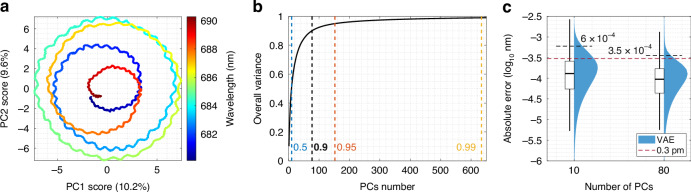
Experimental wavelength prediction for 10 nm broad spectral range. **a** Averaged spectral response in the space of the first two principal components (PCs), where the color spectrum reflects the wavelength changes in the examined band. **b** The dependence of the represented variance in the original data depending on the number of the PCs accounted. Vertical lines represent 50, 90, 95 and 99% levels. **c** Statistics on the wavelength prediction accuracy with VAE augmented experimental dataset comprising 2352 repetitions with the varied number of the features (10, 80)

Comparable performance across a broad spectral range requires ~1000 microresonators, with principal component analysis (PCA) preprocessing used to mitigate growing computational complexity. It transforms the original data into a new set of features (principal components, PCs) that capture the majority of variance. By selecting fewer PCs than microresonators, the method minimizes the input space while preserving the core pattern structure. The averaged broadband pattern in the space of first two PCs (19.8% variance in the original data) is shown in Fig. 6a. Despite the splitting periodicity of wavelength patters, first two PCs show only crossings due to the main FSR. The first 10 PCs represents 50% of variability in the original data, 80 PCs - 90%, 150 PCs - 95% and 630 PCs - 99 (Fig. 6b). We defined 80 PCs as sufficient to represent a chip with 5563 microspheres, while larger number of PCs results in little meaningful information added, but significant effort to adapt the prediction model. Hereby, the broadband experimental dataset 41 × 5563 × 33765 shrinks to 41 × 80 × 33765.

We constructed individual VAEs for most significant PCs (from PC1 to PC10), each reflecting more than 2% of the overall variance and 50% in total. The remaining 70 PCs (overall 40%) have been utilized to train the single VAE with a dataset reshaped by stacking various PCs. Given the limited number of experimental repetitions, we validated the models with 50 VAE outputs generated from averaged experimental response as the input. As a result, all trained VAEs augment 41 experimental measurements to 2352 repetitions. Fig. 6c shows the overall statistics on the wavelength prediction accuracy within the 10 nm broad range. We used the same regression model architecture as previously defined for the narrowband data. For 10 features accounted (50%), the median value for prediction accuracy reaches 100 fm with the 95% quantile at 0.6 pm. With 80 PCs (90%), it improves insignificantly, but each wavelength became unambiguously identified with a specified resolution of 0.3 pm within 80 *μ*s (pre-trained model). Thus, the accuracy gain from 10 to 80 PCs is less pronounced than previously shown between 10 and 80 resonators within 1.2 nm spectral window (Fig. 5a). This opens up the prospect of reduction of the PCs number down to the first 10 which, in turn, allows for extension of the wavelength prediction region up to ~100 nm by keeping the computational efforts to build the regression model at the same level.

## Discussion

Resonance mode splitting by controllable deforming of spherical microcavities enables high-precision and broadband speckle-free reconstructive wavemeter. The proposed novel concept integrates thousands of deformed microspheres on a single chip and provides a unique wavelength-dependent pattern. A set of 100 microspheres with optimal eccentricity level is sufficient to guarantee wavelength prediction accuracy at the intrinsic sensitivity level (300 fm) within the range of ~nm. Such intrinsic sensitivity approaches the ultimate levels demonstrated so far for an integrating sphere as a source for wavelength-dependent speckles. In contrast to the latter, the set of deformed microspheres is superior for miniaturization and enables chip-scale integrability. This can be achieved by aligning dimensions and integrating the WGM chip directly with the camera sensor. The proposed instrument was validated for broadband wavelength estimation using response densification via dimensionality reduction, data augmentation with a variational autoencoder, and ensemble boosting as a regression model. ML interpreter generates output in ≤100 *μ*s and ensures accuracy at the ~100 fm level within ~10 nm, with the potential for its expansion up to ~100 nm in the visible range. Measurement speed is defined by the detector clock, reaching ~kHz in modern systems.

The instrument requires single-run calibration with a tunable laser, but the periodicity of individual microresonator responses allows for a narrower calibration range than the operating range. Similarly to the multimode fiber^45^ or integrating sphere^22^ based configurations, the demonstrated precision limit can be further lowered by more than six orders of magnitude below the intrinsic sensitivity level. This requires extracting latent variables from the raw wavelength pattern within the limited spectral window. PCA used for the broadband prediction can also be applied to diminish the correlation between the closely spaced wavelengths. Therefore, the precision limit for the proposed approach can be moved toward sub-fm level by using an advanced configuration of the calibration light source such as laser locked to the Rubidium line. Supplemented by acousto-optic modulator, it can provide a controlled wavelength shift within ~ 10 fm and with minimal steps at ultimate several attometer level.

## Materials and methods

### Multiplexed microresonator chip

We utilized commercially available soda-lime glass microspheres (Cospheric LLC) and polymethyl methacrylate (PMMA) microspheres (MicroParticles GmbH) with diameters ranging from 50 to 120 *μ*m. They underwent a rigorous cleaning procedure to reduce scattering losses and were then allocated on the 150 *μ*m glass substrates within the area of 10 × 10 mm. Relative dimensions of the microresonators and the monitoring area allow allocation of up to several thousands of microcavities (see Fig. S4 in Supplementary Information). The microspheres are bonded with the substrate via low refractive index films out of the MY-133MC (MyPolymer) material. The latter is deposited onto the substrate and spin-coated to form a uniform film. Until it remains soft, the microspheres are placed by free fall and become partially embed into the film. The film thickness of 2 *μ*m is deemed the minimum necessary to guarantee the robustness of the microresonators-substrate bond. In order to minimize the coupling loss for the microcavities and thus maximize the loaded Q-factor, a separate spacer layer is required to keep the microspheres away from the substrate. To maintain consistent distancing between the microresonators and the substrate within several hundred nanometers, the original water-matched solution was strongly diluted with the solvent. It has been determined that the spacer layer of ≈400 nm enables to observe loaded Q-factor up to ~10^6^. Finally, the substrate with immobilized microcavities is covered by the layer of fluoropolymer CYTOP (AGC Inc.) with a refractive index of 1.34. CYTOP layer was deposited by multiple spin-coating steps on the substrates to guarantee effectively a single medium around microspheres and avoid their contamination.

### Instrument

The measuring chip with embedded microspheres is placed onto the optical prism using immersion oil to maintain stable coupling conditions. A monochrome high-speed global shutter camera (CB262RG-GP-X8G3, Ximea) equipped with lens objective enables signal collection along with spatial distinction of radiated signals from individual microspheres (see Fig. S5 in Supplementary Information). Camera operates at ≈140 fps with field of view reduced to 1.4MP. To equalize the excitation conditions across the microspheres, the chip is illuminated within a collimated beam confined to ≈8 mm, as determined by the achromatic optical collimation package (60FC-T-4-M40-24, Schaefter+Kirchhoff). The elongated collimated beam along the propagation direction at the prism excitation surface is compensated by transforming the laser beam profile from circular to elliptical. To minimize the impact of thermal fluctuations the prism-chip assembly is thermostabilized with a TEC controller (TEC-1091, Meerstetter). Calibration measurements are performed with a tunable diode laser (Velocity, New Focus) within [680:690] nm range directed with a single-mode fiber with controlled polarization. Fizeau configuration-based wavemeter with an absolute accuracy of 30 MHz (WS7-30, HighFinesse) has been utilized as a reference instrument.

### Wavelength pattern collection

In order to match the variations of the radiated intensities with the particular microresonators on the chip, a map of the microcavities is established. This is regarded as a computer vision task where numerous circular objects are identified with the circular Hough Transform. The preprocessing steps involve image denoising, sharpening to enhance edge visibility, background correction, and histogram equalization. An optimized run achieves the detection accuracy of over 95%, with a slight decrease in accuracy attributed to the Hough Transform’s tendency to underestimate. Further details on microspheres mapping can be found in ref. ^40^.

The radiated light integrated over the whole microsphere area is considered the microcavity signal. The experimental set of patterns has been constructed by tuning the laser wavelength over the predefined range multiple times with simultaneous tracking of the intensity distribution over the chip. The laser tuning speed and the camera frame rate have been adjusted to ensure both a reasonable acquisition time to get a single spectrum and a spectral resolution ~100 fm, which is at least twice as much as the absolute accuracy of the calibration wavemeter. Finally, to harmonize the spectral data between the measurements, they are aligned to a common wavelength grid with a step of 300 fm.

### Wavelength pattern simulation

Rigorous evaluation of the reconstructive wavemeter performance requires multiple simulated wavelength patterns for each level of eccentricity (*e*). We proposed the model based on an adapted Voigt profile to account for spectral non-degeneracy of the WGM which arises from eccentricity (see Sec. S2). We varied eccentricity level in the range [5 × 10^−4^, 6.4 × 10^−2^] and included an additional case of *e* = 1 × 10^−6^ to represent nearly ideal spherical symmetry. Each of the simulated sets of patterns contains WGM responses of 200 microresonators with pre-defined positions of the main modes with Q-factors selected from [2 × 10^5^, 2 × 10^6^] and allocated with *FSR*1 = [0.8; 1.2]. The coupled light power for the main mode (${P}_{0}^{m}$) has been searched in [0.125; 1] and *η*_*m*_ has been fixed at 0.8. To maintain simulation time reasonable, we limited the spectral width of the simulated data to 1.2 nm and set the spectral resolution to 0.3 pm. Each WGM spectrum has been simulated 2352 times to consider the impact of the spectral noise. The thermoelastic and thermorefractive coefficients were set at 1 × 10^−4^*K*^−1^ and − 2 × 10^−4^*K*^−1^, respectively. The spectral window for the splitted modes *w* was selected from the range [0.2, 0.7]. For computational reasons, the number of the modes within *w* was limited to 40.

## Supplementary information

Supplementary material for Mode splitting in optical microcavities for speckle-free wavelength reconstruction

## Data Availability

The data that support the findings of this study are available from the corresponding author on reasonable request.
