# Supplementary material for: Mode splitting in optical microcavities for speckle-free wavelength reconstruction
Source: Light Sci Appl. 2026 Jan 1;15:14. doi: 10.1038/s41377-025-02073-9 (PMC12756270; doi:10.1038/s41377-025-02073-9)
Supplement: Supplementary file 1 — Supplementary material for Mode splitting in optical microcavities for speckle-free wavelength reconstruction [file 41377_2025_2073_MOESM1_ESM.pdf]

Supplementary Information for:

Mode splitting in optical microcavities for speckle-free wavelength reconstruction

Ivan Saetchnikov[1], Elina Tcherniavskaia[2], Andreas Ostendorf[3], Anton Saetchnikov[3]

[1]Radio Physics Department, Belarusian State University, Minsk, 220064, Belarus

[2]Physics Department, Belarusian State University, Minsk, 220030, Belarus

[3]Chair of Applied Laser Technologies, Ruhr University Bochum, Bochum, 44801, Germany

Corresponding author(s): Anton Saetchnikov. E-mail(s): anton.saetchnikov@rub.de

S1 Eccentricity splitting in deformed microspheres

The spectra of two representative glass microspheres with mode splitting are provided in Fig. S1. Both spectra shown in Fig. S1 are characterized by the presence of plurality of the splitted modes of different mean loaded Q-factors (5.3×10^5 and 1.1×10^5) that exist within the splitting windows of comparable width. Difference in size of the microcavities that manifests as different azimuthal free spectral range ($FSR1$) leads to different frequency of splitting blocks occurrence within the same spectral range. Here, splitting block is shaped by the loaded Q-factor and the spacing between the splitted modes ($FSR2$). Similar pitch value for splitted modes (20.8 and 16.8 pm) in case of lower Q-factor (1.1×10^5) results in partial overlapping of the resonance lines and formation of the broad resonance profile (Fig. S1d). It is still characterized by high-frequency intensity modulation due to mode splitting, which decreases in severity with increase of the spectral overlapping of the splitted modes. As a result, frequent splitting that appears for slightly deformed cavities and insufficiently high Q-factor can lead to the transformation of the splitted spectrum into low Q-factor resonance profile. Thus, efficient generation of splitted spectrum is possible when the optimal ratio between Q-factor and deformation of the microresonator is ensured.

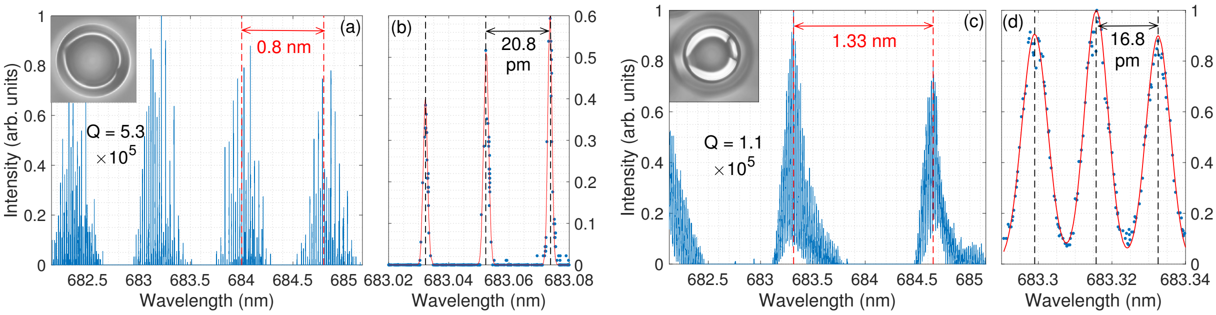

Fig. S1: WGM spectra with eccentricity mode splitting for two representative glass microspheres of different dimensions. The spectra over several free spectral ranges (FSR) along with zoom into the splitting region for bigger and smaller microcavities (a), (b) and (c), (d), correspondingly. Blue lines and dots represent experimental results, red lines - fitting of the resonances with a Voigt profile. Insets provide the microscope picture of the microresonators highlighting their differences in size.

As a result of sphere deformation, the ellipsoid surface with different semi-major and semi-minor axes is obtained. Thus, the orientation of the excitation beam plane with respect to these axes will define the effective eccentricity degree. To demonstrate its reliance and mode splitting mechanics in the WGM spectrum, we rotated the chip relative to the laser beam propagation plane. Thereby, different eccentricity levels along the WGM propagation path for the distorted microspheres on the chip can be realized. Variations of the mode splitted specta for a representative deformed microcavity for four different orientations of the chip relative to the beam are presented in Fig. S2.

The results show clear alterations in the mode splitting when the excitation plane is varied. In particular, by increasing the rotation angle (entirely defined by selection of the initial) the window for mode splitting expands and eventually takes up the entire FSR of the WGM. In the demonstrated example, the $FSR2$ changes from 8 to 65 pm when different orthogonal planes in the microresonator are utilized to excite the WGM. Under consideration that $FSR1$ for the depicted microcavity accounts ≈ 0.8 nm, the effective eccentricity level changes from 1% to 8%. It has been revealed that the splitting step coincides for modes of different orders. Positions of the neighboring splitted modes of different orders are marked as vertical lines of different colors in Fig. S2. Severe deformations of the selected microsphere that manifests with up to 8% eccentricity level explains the non-uniformity of the splitting step variations with changes

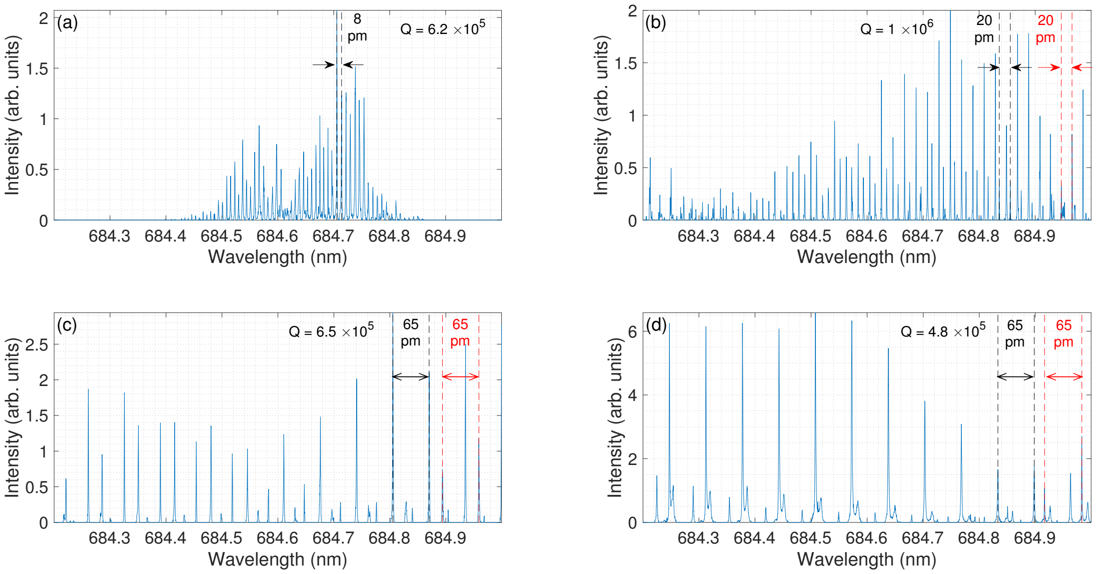

Fig. S2: Mode spitted spectra in a deformed glass microsphere at different chip orientations relative to the excitation plane. Orthogonal excitation planes in the representative glass optical microresonator 0° (a) and 90° (d) along with two states between them 20° (b) and 66° (c) are shown. Black and red colored vertical lines indicate modes of different orders.

of the microcavity orientation. In particular, by changing the orientation of the microsphere on 24° (from 66° to 90°) the same 65 pm splitting step was revealed. This indicates a location near the projection with maximum distortion. Comparable rotation of microresonator on 20° (from 0° to 20°) results in more than doubling of the splitting step. Besides the splitting step, the orientation of the microresonator affects the loaded Q-factor where for particular orientation it reaches 1×10^6. It is, however, weakly related to the eccentricity level and is rather determined by the scattering losses in the plane of the mode propagation.

S2 Model of the eccentricity splitted WGM spectra

To study the impact of the eccentricity-induced mode splitting on the degenerated WGM spectrum for high-precision wavemeters, a dataset with well-controlled conditions for eccentricity splitting is necessary. While rigorous mathematical models for WGM resonances in optical microresonators are well established and can be found elsewhere, the multiplexed microresonator imaging configuration and eccentricity-induced mode splitting require adaptation to the original WGM model. The Lorentzian broadening mechanism characterizes the WGM spectrum of ideal microresonator with degenerated modes. Natural shape alterations, material inhomogeneity, roughness on the microresonator surface and glass substrate lead to additional inhomogeneous (Gaussian) broadening [46]. The convolution of the Gaussian and Lorentzian profiles is known in spectroscopy as Voigt profile and can be used to describe WGM spectral patterns [47]. This can be simplified to the pseudo-Voigt form, which describes power spectrum of the microcavities ($P(\omega)$) as follows:

$$P = \sum_{i=1}^{n} P_0^i \Big[\eta_i \Big(\frac{(\gamma_i/2)^2}{(\omega - \omega_0^i)^2 + (\gamma_i/2)^2} \Big) + \\ + (1 - \eta_i) \exp\Big(\frac{-(\omega - \omega_0^i)^2}{{\gamma_g^i}^2} \Big) \Big] \quad \text{(S1)}$$

where n is the number of modes, P_0^i is the coupled light power of the ith mode, η_i characterizes the ratio between the homogeneous and inhomogeneous broadening mechanisms for the ith mode, ω_0^i is the central frequency of the ith mode, γ_i is the full width at half maximum (FWHM) of the ith mode, and $\gamma_g^i = \gamma_i/(2\sqrt{2\ln 2})$.

Initially, the degenerated spectrum with one (main) mode allocated within the cavity's free spectral range ($FSR1$) is simulated. Its spectral position is influenced by a range of independent factors, including resonator parameters (such as size, shape, and material), light coupling settings, and illumination polarization. Therefore, the distribution of the resonance position for degenerated mode (ω_0^m) among the cavities is assumed to be uniform within the $FSR1$. The latter parameter varies for the cavities due to their feature differences and is assumed to follow uniform distribution in the range [$FSR1^{\min}$, $FSR1^{\max}$]. Similarly, the Q-factors of the modes are also anticipated to be uniformly distributed between the minimum ($Q_{\text{tot}}^{\min}$) and maximum ($Q_{\text{tot}}^{\max}$) observable values. The coupled light power for the main mode (P_0^m) is also defined as uniformly distributed number from $P_0^{\min}$ to $P_0^{\max}$. When the simulation region exceeds the $FSR1$, main modes with different azimuthal numbers are simulated by allocating them equidistantly with $FSR1$ around ω_0^m.

When the azimuthal mode degeneracy is lifted, the mode splitting shows up. In order to simulate this, we have introduced two parameters to the model. Eccentricity level ($e = \frac{a-b}{a}$), where a and b are the semi-axes, describes the nonsphericity of the microresonator. This parameter is used to define the free spectral range of the the mode splitting as ($FSR2 = eFSR1$) [37, 38]. Another parameter is the window for mode splitting (w) that indicates the spectral region for phase matched energy transfer from the prism to the microresonators. Within the window w a set of the equidistantly ($FSR2$) allocated central frequencies ω_0^i around ω_0^m has been defined. Under the assumption of small deviations from the spherical shape, the resonance line widths of the side modes (ω_0^i) are not expected to deviate considerably from the main mode (ω_0^m) and, therefore, are set to be identical to the main mode. The same is done for ratios between homogeneous and inhomogeneous broadening (η_i) where all of them are set equal to η_m. Coupled light power for the modes within the splitting window (P_0^i) are expected to follow normal distribution (phase matching conditions degrade with moving away from the central mode) with the maximum at previously defined P_m^i. In order to ensure variability among different microresonators, the set P_0^i is defined as the histogram distribution obtained for the randomly generated values. The procedure repeats for other main modes with different azimuthal numbers to obtain the spectrum exceeding $FSR1$. As a result, a group of the following parameters ω_0^i , γ_i, P_0^i, η_i is established and noise-free WGM spectrum with lifted degeneracy can be simulated using Eq. S1.

Real spectrum is accompanied by various noise components of both fundamental and technical nature. The impact of the fundamental noise sources depends on the integration time, where noise due to temperature variations becomes more prominent than the shot noise for time scales longer than a few milliseconds. This aligns with the minimum possible averaging time defined by the maximum camera frame rate of the multiplexed microresonator imaging scheme. Technical noise sources are linked to the laser and detector (camera). Laser instabilities, such as wavelength sweeping region repeatability and output power fluctuations, are considered negligible. The laser operates within a spectral range with no notable power fluctuations, and collected spectra are normalized to suppress long-term power variations. Camera-generated noise, described as Gaussian noise, is dominated by read-out noise in the selected operation configuration. As a result, thermoelastic, thermorefractive, and detector noise contributions must be accounted for in the eccentricity splitted WGM spectrum model.

The instability of the resonance central line $\sigma\omega_0^i$ induced by the temperature fluctuations σT through the thermorefractive ($\alpha_n = \frac{dn_{eff}}{dT}$) and thermoelastic effects ($\alpha_R = \frac{1}{R}\frac{dR}{dT}$) is described by equation:

$$\frac{\sigma\omega_0^i}{\sigma T} = -\frac{1}{n_{eff}}\alpha_n\omega_0^i - \alpha_R\omega_0^i \tag{S2}$$

For each frequency ω in the spectrum that is taken frame-wise by the camera while the laser is being swept, the positions of the mode central frequencies are represented as $\omega_0^i + \sigma\omega_0^i$. Despite the discrepancy in the effective refractive index n_{eff} among the modes, the modes are assumed to be entirely confined

within the cavity, making n_{eff} equivalent to the resonator refractive index n_r. Short-term temperature fluctuations have been studied experimentally and determined to follow a normal distribution with a standard deviation of 0.01 K. The impact of thermal fluctuations on the WGM linewidth was analyzed for scattering Q_{scat} and coupling (Q_{coup}) limited Q-factors. The radiation Q_{rad} and absorption Q_{abs} limited Q-factors attain 10^{15} and 10^7, respectively, which exceed the experimentally observed values for the loaded Q-factors (up to 10^6). By using the expressions for Q_{scat} and Q_{coup} proposed in [48, 49] the Q-factor instabilities are calculated to not exceed $\sim 10^0$ for the mentioned above level of temperature variations (0.01 K). For this reason, the impact of the temperature fluctuations on the linewidth of the WGMs is omitted in the model. The Gaussian noise representing the camera's read-out noise is generated with the mean value equal to zero. The standard deviation of the noise is estimated from the experimental data by examining signal variations under non-resonant conditions and equals to ≈ 0.001.

S3 Regression interpreter model

Wavelength prediction was treated as a regression task on tabular data, that can be solved by decision-tree-based (DTb) and deep learning approaches (dNN, deep neural network). The detector noise has a vertical projection into the spectrum, whereas the thermal noise has a horizontal one. This presents an additional challenge to data preprocessing, which, in turn, makes dNN less suitable for wavelength prediction. Furthermore, since the features (i.e., microcavities in this case) are essentially equivalent, selecting the most prominent ones for the training dataset results in the lack of generalization due to unique resonator spectra. Given that dNN are more susceptible to inefficient features than DTb models, the latter are deemed to be the optimal baseline approach.

Ensemble boosting methods like LightBoost, XGBoost, and CatBoost are highly accurate and robust, especially for tabular data, and are categorized as DTb methods. Gradient boosting (GB) models differ from other ensemble methods like bagging and stacking by building decision trees sequentially, where each tree corrects errors from the previous ones. With well-tuned parameters, this iterative process enables GB models to capture complex patterns efficiently. LightBoost represents a modification of GB that integrates the "Gradient-based One Side Sampling" and "Exclusive Feature Bundling modules". The first module focuses on training examples that yield larger gradients, thereby accelerating training and reducing the computational complexity. The second module employs a boosting approach that combines sparse (mostly zero) mutually exclusive features by bagging these attributes. This enables smart feature selection, which is particularly useful for wavelength prediction, where preliminary feature selection is restricted. These modules make LightGBM both accurate and fast, with improved memory efficiency and scalability over the slower XGBoost. CatBoost can attain enhanced regression accuracy bit for datasets with categorical features.

The tuning process was dedicated to determine the optimal number of boosting iterations with its maximum set to 1000. Learning rate, number of leaves, maximum depth of the decision tree, and L1 and L2 regularization parameters were optimized based on the results for a 5-fold cross-validation. It ensures comprehensive coverage of all available wavelengths and thus enhances the tuning relevance. The tree is constructed until it either reaches the maximum depth or is unable to identify an optimal split. By establishing this parameter, the model is prevented from fitting noise in the training data with excessive precision, thereby reducing the probability of overfitting. In order to enhance the robustness of the model, the number of leaves is set to a value that is considerably below two raised to the power of the maximum depth. By increasing the maximum depth and the leaves number, the model performance can be improved by the cost of higher probability for overfitting and time required for training. To achieve trade-off between these elevated parameter values, we introduced the minimum data-in-leaf parameter, which establishes the minimum number of observations required in a leaf node. Low value of this parameter may result in overfitting, as excessively specific leaf nodes may capture noise and thereby reduce generalization. Conversely, an excessively high value may result in underfitting. To further mitigate the risk of overfitting, we employed regularization using L1 and L2 parameters. The training process with the predefined learning rate continues until either the maximum number of boosting iterations was reached or the performance

on the validation test does not improve during the last 50 iterations. Lightboost regression model is built and trained using the following hardware configurations: 14 cores Intel Xeon W-2275 processor, 128 GB of RAM coupled with Nvidia T4 GPU with 16 GB of RAM as well as Nvidia Quadro RTX 6000 with 24 GB of RAM for training and optimizing models. The processing was conducted using the Python programming language, which leveraged the CUDA and PyTorch libraries, along with a set of additional Python libraries, including TensorFlow and Keras.

S4 VAE model for pattern conditioning

Large volume of high-quality data is essential for achieving robust and accurate predictions. This is the common challenge in many data-driven inverse problems since collection for such data is a major time-consuming constraint. Also, ensuring the data quality is particularly difficult, as variations in physical constants and experimental conditions can introduce errors and biases, which, in turn, negatively affect the performance of the predictive model. There are two competing approaches to address this challenge. The first involves utilization of the analytical model to generate sufficient data, pre-training the predictive model on this simulated dataset, and then fine-tuning it using a limited set of experimental results. However, given the enormous spectral variability among WGM microcavities, including the presence of multiple modes of various orders and mode splitting, the generated multiplexed spectral data may suffer from assumptions in the WGM spectrum model. A variational autoencoder (VAE)-based architecture has been put forth as a potential solution for data augmentation in the context of limited experimental spectra. The VAE core is two mirrored dNNs referred to as encoder and decoder. The first one transforms input data into a lower-dimensional latent space, the second reconstructs the original data from the latent space. In order to achieve a one-step data augmentation, we propose the inclusion of one-dimensional convolutional layers for both the encoder and the decoder. An alternative to the convolutional is the use of long short-term memory (LSTM) layers, which can be effectively employed for limited spectral width, thereby avoiding the risk of losing long-term information when processing long spectra. This requires the spectral windows from the original sequence to be sampled, trained and merged to obtain the full spectrum.

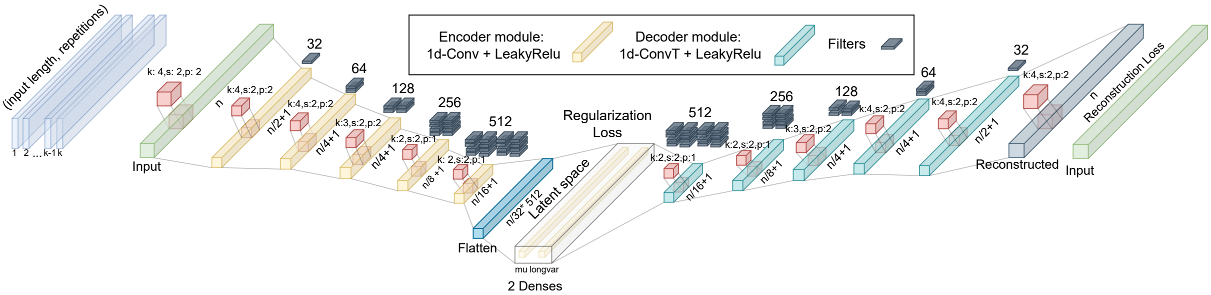

Fig. S3: Architecture of the variational autoencoder for data augmentation of the multiplexed WGM spectral data.

Encoder comprises five modules each out of a convolutional layer followed by a LeakyRelu activation function (Fig. S3). The number of filters is increased by a factor of two at each successive layer, with filter sizes of 32, 64, 128, 256, and 512. The sizes of the kernel, stride, and padding were optimized in order to achieve a continuous halving of the input size sequence (n) at each step, reducing it from n to $n/32$. During the compression process on the convolutional layers, the size of the convolutional kernel has been reduced in order to facilitate the manipulation of local patterns and the extraction of fine-grained patterns in the data. Convolution modules are followed by two fully connected layers in a latent space with a dimension of 2000. The decoder consists of five up-sampled modules of transposed convolutional layers

with LeakyReLU activations, featuring filter sizes of 512, 256, 128, 64, and 32. The parameters for kernel size, padding, and stride have been configured in accordance with that defined before for the encoder. In particular, the first convolutional layer in the encoder employs the identical parameters as the final deconvolutional layer in the decoder, and so forth. The VAE is trained using the Adam optimizer with an adaptive learning rate of 1×10^{-3} decreasing each 50 epochs on gamma 0.1. The objective is to minimize a loss function comprising two components: the reconstruction loss, based on the cumulative mean squared error (MSE) across all batch sequences, and the regularization loss, derived from the Kullback-Leibler divergence (KLD). The parameter beta, which adjusts the contribution of the KLD term in the total loss, is set to 0.005. The training process of the VAE lasts for 150 epochs, continuously updating the trained model with weights that demonstrate the best performance on the testing set until the total training loss reaches a plateau. VAE model is optimized using the same hardware configuration as utilized for the regression model.

S5 Physical model implementation

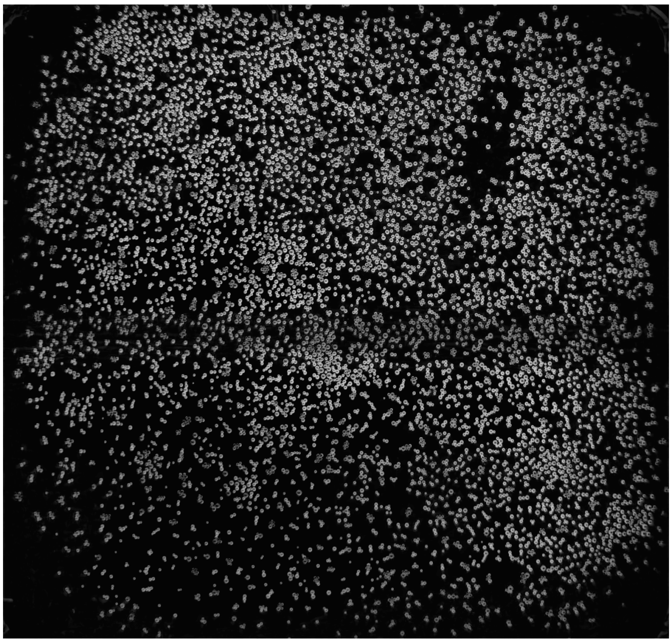

Fig. S4: Overview of the multiplexed microresonator chip with PMMA microspheres. Demonstrated image is captured by the measuring instrument and is processed for denoising and background glow removal procedures to enhance clarity.

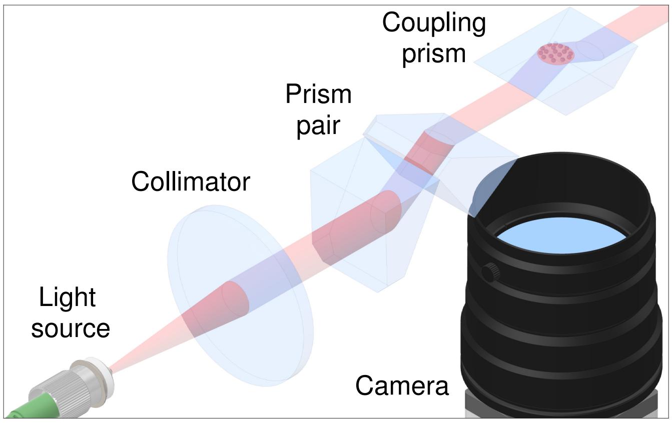

Fig. S5: Multiplexed microresonator imaging method for wavemeter with eccentricity-splitted microspheres.

References

[1] Udem, T., Holzwarth, R. & Hänsch, T. W. Optical frequency metrology. *Nature* **416**, 233–237 (2002).

[2] Couturier, L. *et al.* Laser frequency stabilization using a commercial wavelength meter. *Review of scientific instruments* **89**, 043103 (2018).

[3] Newman, Z. L. *et al.* Architecture for the photonic integration of an optical atomic clock. *Optica* **6**, 680–685 (2019).

[4] de Leon, N. P. *et al.* Materials challenges and opportunities for quantum computing hardware. *Science* **372**, eabb2823 (2021).

[5] Jørgensen, A. A. *et al.* Petabit-per-second data transmission using a chip-scale microcomb ring resonator source. *Nature Photonics* **16**, 798–802 (2022).

[6] Dobosz, M. & Kożuchowski, M. Overview of the laser-wavelength measurement methods. *Optics and Lasers in Engineering* **98**, 107–117 (2017).

[7] Coluccelli, N. *et al.* The optical frequency comb fibre spectrometer. *Nature communications* **7**, 12995 (2016).

[8] Niu, R. *et al.* khz-precision wavemeter based on reconfigurable microsoliton. *Nature communications* **14**, 169 (2023).

[9] Xu, B. X. *et al.* Whispering-gallery-mode barcode-based broadband sub-femtometer-resolution spectroscopy with an electro-optic frequency comb. *Advanced Photonics* **6**, 016006 (2024).

[10] Gardner, J. L. Compact fizeau wavemeter. *Applied optics* **24**, 3570–3573 (1985).

[11] Rotter, S. & Gigan, S. Light fields in complex media: Mesoscopic scattering meets wave control. *Reviews of Modern Physics* **89**, 015005 (2017).

[12] Yang, Z. Y. *et al.* Miniaturization of optical spectrometers. *Science* **371**, eabe0722 (2021).

[13] Wan, N. H. *et al.* High-resolution optical spectroscopy using multimode interference in a compact tapered fibre. *Nature communications* **6**, 7762 (2015).

[14] Bruce, G. D. *et al.* Femtometer-resolved simultaneous measurement of multiple laser wavelengths in a speckle wavemeter. *Optics Letters* **45**, 1926–1929 (2020).

[15] Gao, Z. *et al.* Breaking the speed limitation of wavemeter through spectra-space-time mapping. *Light: Advanced Manufacturing* **4**, 187–194 (2024).

[16] Wang, T. L. *et al.* Decoding wavelengths from compressed speckle patterns with deep learning. *Optics and Lasers in Engineering* **180**, 108268 (2024).

[17] Redding, B. *et al.* Compact spectrometer based on a disordered photonic chip. *Nature Photonics* **7**, 746–751 (2013).

[18] Wang, Z. *et al.* Single-shot on-chip spectral sensors based on photonic crystal slabs. *Nature communications* **10**, 1020 (2019).

[19] Brown, C. *et al.* Neural network-based on-chip spectroscopy using a scalable plasmonic encoder. *ACS nano* **15**, 6305–6315 (2021).

[20] Sun, Q. *et al.* Compact nano-void spectrometer based on a stable engineered scattering system. *Photonics Research* **10**, 2328–2336 (2022).

[21] Metzger, N. K. *et al.* Harnessing speckle for a sub-femtometre resolved broadband wavemeter and laser stabilization. *Nature communications* **8**, 15610 (2017).

[22] Gupta, R. K. *et al.* Deep learning enabled laser speckle wavemeter with a high dynamic range. *Laser & Photonics Reviews* **14**, 2000120 (2020).

[23] Facchin, M. *et al.* Determining intrinsic sensitivity and the role of multiple scattering in speckle metrology. *Nature Reviews Physics* **6**, 500–508 (2024).

[24] Popoff, S. M. *et al.* Measuring the transmission matrix in optics: An approach to the study and control of light propagation in disordered media. *Physical Review Letters* **104**, 100601 (2010).

[25] Kwak, Y. *et al.* A pearl spectrometer. *Nano letters* **21**, 921–930 (2021).

[26] Edrei, E. *et al.* Chip-scale atomic wave-meter enabled by machine learning. *Science advances* **8**, eabn3391 (2022).

[27] Braginsky, V. B., Gorodetsky, M. L. & Ilchenko, V. S. Quality-factor and nonlinear properties of optical whispering-gallery modes. *Physics Letters A* **137**, 393–397 (1989).

[28] Vahala, K. J. Optical microcavities. *Nature* **424**, 839–846 (2003).

[29] Loyez, M. *et al.* From whispering gallery mode resonators to biochemical sensors. *ACS sensors* **8**, 2440–2470 (2023).

[30] Tang, S.-J. *et al.* Single-particle photoacoustic vibrational spectroscopy using optical microresonators. *Nature Photonics* **17**, 951–956 (2023).

[31] Zossimova, E. *et al.* Whispering gallery mode sensing through the lens of quantum optics, artificial intelligence, and nanoscale catalysis. *Applied Physics Letters* **125**, 030501 (2024).

[32] Wan, Y. Y. *et al.* Reconstructive spectrum analyzer with high–resolution and large–bandwidth using physical–model and data–driven model combined neural network. *Laser & Photonics Reviews* **17**, 2201018 (2023).

[33] Schweiger, G., Nett, R. & Weigel, T. Microresonator array for high-resolution spectroscopy. *Optics Letters* **32**, 2644–2646 (2007).

[34] Petermann, A. B. *et al.* Surface-immobilized whispering gallery mode resonator spheres for optical sensing. *Sensors and Actuators A: Physical* **252**, 82–88 (2016).

[35] Berkis, R. *et al.* Wavelength sensing based on whispering gallery mode mapping. *Fibers* **10**, 90 (2022).

[36] Sumetsky, M. Whispering-gallery-bottle microcavities: The three-dimensional etalon. *Optics Letters* **29**, 8–10 (2004).

[37] Gorodetsky, M. L. & Fomin, A. E. Geometrical theory of whispering-gallery modes. *IEEE Journal of Selected Topics in Quantum Electronics* **12**, 33–39 (2006).

[38] Ilchenko, V. S. *et al.* Whispering gallery mode diamond resonator. *Optics Letters* **38**, 4320–4323 (2013).

[39] Xie, Y. *et al.* Batch fabrication of high-quality infrared chalcogenide microsphere resonators. *Small* **17**, e2100140 (2021).

[40] Saetchnikov, A. V. *et al.* Reusable dispersed resonators-based biochemical sensor for parallel probing. *IEEE Sensors Journal* **19**, 7644–7651 (2019).

[41] Saetchnikov, A. V. *et al.* Deep-learning powered whispering gallery mode sensor based on multiplexed imaging at fixed frequency. *Opto-Electronic Advances* **3**, 200048 (2020).

[42] Saetchnikov, A. V. *et al.* Intelligent optical microresonator imaging sensor for early stage classification of dynamical variations. *Advanced Photonics Research* **2**, 2170040 (2021).

[43] Saetchnikov, A. V. *et al.* Detection of per- and polyfluoroalkyl water contaminants with a multiplexed 4d microcavities sensor. *Photonics Research* **11**, A88–A96 (2023).

[44] Saetchnikov, A. V. *et al.* Two-photon polymerization of optical microresonators for precise ph sensing. *Light: Advanced Manufacturing* **5**, 624–636 (2024).

[45] Bruce, G. D. *et al.* Overcoming the speckle correlation limit to achieve a fiber wavemeter with attometer resolution. *Optics Letters* **44**, 1367–1370 (2019).

[46] Le Thomas, N. *et al.* Effect of a dielectric substrate on whispering-gallery-mode sensors. *Journal of the Optical Society of America B* **23**, 2361–2365 (2006).

[47] Francois, A. & Himmelhaus, M. Optical sensors based on whispering gallery modes in fluorescent microbeads: size dependence and influence of substrate. *Sensors (Basel, Switzerland)* **9**, 6836–6852 (2009).

[48] Gorodetsky, M. L. & Ilchenko, V. S. Optical microsphere resonators: Optimal coupling to high-Q whispering-gallery modes. *Journal of the Optical Society of America B* **16**, 147–154 (1999).

[49] Gorodetsky, M. L., Pryamikov, A. D. & Ilchenko, V. S. Rayleigh scattering in high-Q microspheres. *Journal of the Optical Society of America B* **17**, 1051–1057 (2000).
